# Site-specific acetylation of ISWI by GCN5

**DOI:** 10.1186/1471-2199-8-73

**Published:** 2007-08-30

**Authors:** Roger Ferreira, Anton Eberharter, Tiziana Bonaldi, Mariacristina Chioda, Axel Imhof, Peter B Becker

**Affiliations:** 1 Adolf-Butenandt-Institut, Molekularbiologie, 80336 München, Germany; 2 European Patent Office – Biotechnology, D-80339 München, Germany; 3 Max-Planck Institut für Biochemie, D-82152 Martinsried, Germany

## Abstract

**Background:**

The tight organisation of eukaryotic genomes as chromatin hinders the interaction of many DNA-binding regulators. The local accessibility of DNA is regulated by many chromatin modifying enzymes, among them the nucleosome remodelling factors. These enzymes couple the hydrolysis of ATP to disruption of histone-DNA interactions, which may lead to partial or complete disassembly of nucleosomes or their sliding on DNA. The diversity of nucleosome remodelling factors is reflected by a multitude of ATPase complexes with distinct subunit composition.

**Results:**

We found further diversification of remodelling factors by posttranslational modification. The histone acetyltransferase GCN5 can acetylate the *Drosophila *remodelling ATPase ISWI at a single, conserved lysine, K753, *in vivo *and *in vitro*. The target sequence is strikingly similar to the N-terminus of histone H3, where the corresponding lysine, H3K14, can also be acetylated by GCN5. The acetylated form of ISWI represents a minor species presumably associated with the nucleosome remodelling factor NURF.

**Conclusion:**

Acetylation of histone H3 and ISWI by GCN5 is explained by the sequence similarity between the histone and ISWI around the acetylation site. The common motif RK^T^/_S_xGx(K^ac^)xP^R^/_K _differs from the previously suggested GCN5/PCAF recognition motif GKxxP. This raises the possibility of co-regulation of a nucleosome remodelling factor and its nucleosome substrate through acetylation of related epitopes and suggests a direct crosstalk between two distinct nucleosome modification principles.

## Background

Disruption of DNA-histone interactions by nucleosome remodelling ATPases may lead to a variety of transitions of chromatin structure, such as the partial or complete disassembly of nucleosomes, the exchange of histones, or the sliding of intact histone octamers on DNA [1-4]. In many cases their activity is focused on local disruption of the nucleosomal fibre through recruitment of DNA-binding regulators to promote access of factors further downstream in the cascade of events that leads to promoter opening [2]. However, genome-wide processes like replication, DNA damage responses or homologous recombination require chromatin to be dynamic on a global scale. In addition to generating local access to nucleosomal DNA, nucleosome disruption may also have profound consequences for the folding of the nucleosomal fibre into higher order structures [5,6].

One largely unresolved issue to date is how the activity of chromatin remodelling enzymes is regulated. Established regulatory principles that govern, for example, metabolic enzymes also apply to nucleosome remodelling ATPases, but our knowledge is still anecdotal. The expression of nucleosome remodelling ATPases may be selective. For example, the fact that the mammalian ISWI isoform SNF2H is abundant in proliferating cells, whereas the related SNF2L is enriched in terminally differentiated neurons points to functional diversification of related remodelling enzymes and specialized roles in proliferation/differentiation control [7]. Such a role has also been suggested for the SWI2/SNF2-type ATPase hBRM by the early finding that its expression is down-regulated when cells receive a mitogenic stimulus or during *ras*-mediated oncogenic transformation, whereas its forced expression partially reverses transformation [8].

A further regulatory strategy involves post-translational modifications of enzymes, such as their phosphorylation. Phosphorylation of hBRM and BRG-1 during mitosis correlates with dissociation of these remodellers from the chromosomes during condensation [9]. Muchardt and colleagues also showed that acetylation of hBRM correlates with a reduced inhibition of cell growth [10]. The possibility of regulating nucleosome remodelling ATPases by lysine acetylation is intriguing given that properties of their substrates, the histones, are most prominently modulated by acetylation at their exposed N-termini [11].

Here we describe another example of potential regulation of a remodelling ATPase by acetylation. We found that *Drosophila *ISWI, the founding member of a family of nucleosome remodelling ATPases, was preferentially acetylated by GCN5 at a single lysine within a amino acid sequence of high similarity to the N-terminus of histone H3. This acetylated lysine corresponds to lysine 14 in histone H3 (H3K14), a known target for GCN5, suggesting that this acetyltransferase may affect the chromatin structure by two distinct strategies: by acetylation of the nucleosomes and by modification of a nucleosome remodelling enzyme.

## Results

### ISWI is acetylated in vivo

**Figure 1:**
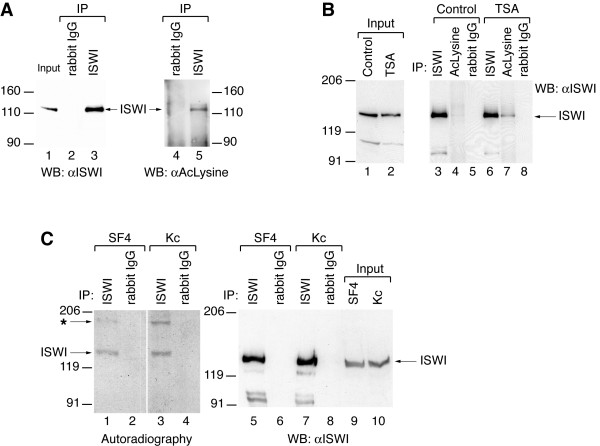
**ISWI is acetylated *in vivo***. (A) ISWI was immunoprecipitated from whole cell extracts of *Drosophila *SF4 cells. A parallel immunoprecipitation (IP) with rabbit IgG antibodies served as specificity control. Enrichment of ISWI was documented by Western blotting (WB) using an αISWI antibody (lanes 1–3). A pan-acetyl-lysine (αAcLysine) antibody detects a protein co-migrating with ISWI (lanes 4, 5). (B) Kc cells were cultured for 17 hrs in the presence or absence (control) of 200 ng/ml trichostatin A (TSA). Whole cell extracts contained equal amounts of ISWI (lanes 1, 2). Proteins were immunoprecipitated from these extracts with αISWI or αAcLysine antibody as indicated. ISWI was detected in the precipitate by Western blot analysis. (C) *Drosophila *SF4 and Kc cells were metabolically labelled with [^3^H]-acetic acid and extracts prepared. Extract ISWI levels were visualised by Western blotting (lanes 9, 10). ISWI was immunoprecipitated from these extracts and visualised by Western blotting (lanes 5–8). An equivalent gel was then autoradiographed (lanes 1–4) in order to detect acetylated protein. The arrow points to the position of ISWI, the asterisk indicates an unknown acetylated protein.

In order to explore whether ISWI was acetylated in *Drosophila *cell lines we immunoprecipitated the ATPase from extracts of SF4 cells (Figure 1A, lanes 1–3). Probing the precipitate with a pan-acetyl-lysine antibody (αAcLysine) we detected a labelled protein migrating at the position of ISWI (Figure 1A, lanes 4, 5). To facilitate detection of acetylated ISWI we treated Kc cells with the histone deacetylase inhibitor Trichostatin A (TSA), prepared whole cell extracts and monitored ISWI levels (Figure 1B, lanes 1, 2). We then immunoprecipitated ISWI or proteins containing acetylated lysines from these extracts. In the absence of TSA ISWI was barely detectable in the αAcLysine precipitate, which may be due to the inefficiency of the antibody and/or the small amounts of acetylated ISWI present in Kc cells (Figure 1B, lanes 3–5). However, upon overnight TSA treatment the levels of acetylated ISWI increased significantly (Figure 1B, lanes 6–8). Taken together these results suggested that a minor fraction of ISWI was acetylated in Kc and SF4 cells. In order to confirm this notion by an independent experiment we metabolically labelled SF4 and Kc cells by addition of [^3^H]-acetic acid for 3 hrs to the growth medium, prepared extracts and determined the ISWI levels as before (Figure 1C, lanes 9, 10). ISWI was immunoprecipitated from these extracts (Figure 1C, lanes 5–8) and acetylated proteins in the precipitate were detected by gel electrophoresis and autoradiography. A band migrating at the position of ISWI was only detectable in the αISWI precipitate, but was absent in the control (Figure 1C, lanes 1–4). A second labelled band points to an acetylated ISWI-associated protein of unknown identity (asterisk in Figure 1C). Collectively, these data demonstrate that a relatively minor fraction of ISWI is acetylated in *Drosophila *tissue culture cells.

### Histone acetyltransferases with substrate preference for the histone H3 N-terminus acetylate ISWI in vitro

**Figure 2:**
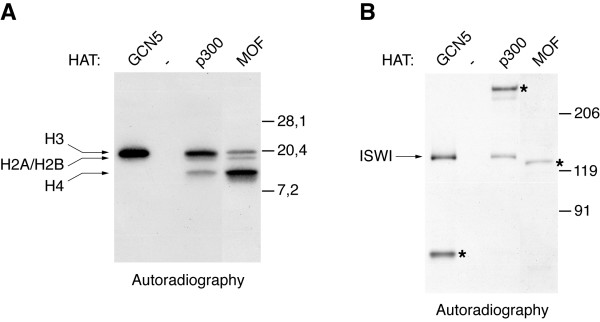
***In vitro*, ISWI is specifically acetylated by HATs able to modify histone H3**. (A) The histone acetyltransferase activities of recombinant GCN5, p300 and MOF were assayed in reactions containing all four histones. Incorporation of [^3^H]-acetyl-coenzyme A leads to labelled proteins, which are separated by SDS-PAGE and detected by autoradiography of the gel. Bands corresponding to acetylated histones are marked by arrows. (B) Recombinant ISWI was incubated in acetylation reactions with GCN5, p300 or MOF. Acetylated ISWI (arrow) was detected as in (A). All other bands (asterisks) are due to auto-acetylation of the corresponding enzyme.

We wished to identify acetyltransferases able to acetylate ISWI. Towards this end we expressed and purified GCN5, p300 and MOF and confirmed their activity and specificity on histone substrates. In agreement with published work [12-14], GCN5 only acetylated histone H3, p300 mainly acetylated H3 and to a lesser extent H4 and MOF preferred histone H4 as a substrate (Figure 2A). Replacing the histone substrate with recombinant ISWI we found that GCN5 and p300 were also able to acetylate ISWI, whereas MOF did not (Figure 2B). The activity of all HATs in these assays is conveniently controlled by their autoacetylation (bands labelled with asterisks in Figure 2B). Interestingly, the ability to acetylate ISWI correlated with the enzymes' preference for H3 among the histones. Because acetylation of ISWI by GCN5 was particularly robust, we employed GCN5 in the subsequent analyses.

### GCN5 acetylates ISWI at lysine 753

**Figure 3:**
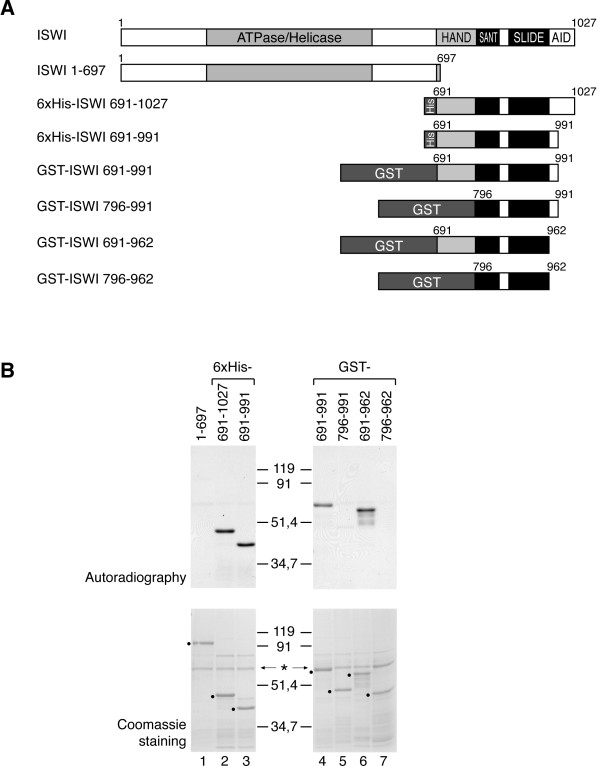
**ISWI is acetylated in the HAND domain**. (A) Schematic representation of ISWI, the extent of known domains (first line) and of deletion mutants used for mapping the acetylation site. Numbers correspond to the first and the last amino acids of the proteins. They were expressed in *E. coli *with either HIS_6_- or glutathione-S-transferase- (GST-) tags. (B) The different ISWI fragments displayed in (A) were acetylated *in vitro *with GCN5. Reactions were resolved by SDS-PAGE and visualized by autoradiography (upper panel) and Coomassie staining (lower panel). Black dots label the positions of the ISWI fragments. An asterisk marks the band corresponding to GCN5.

**Figure 4:**
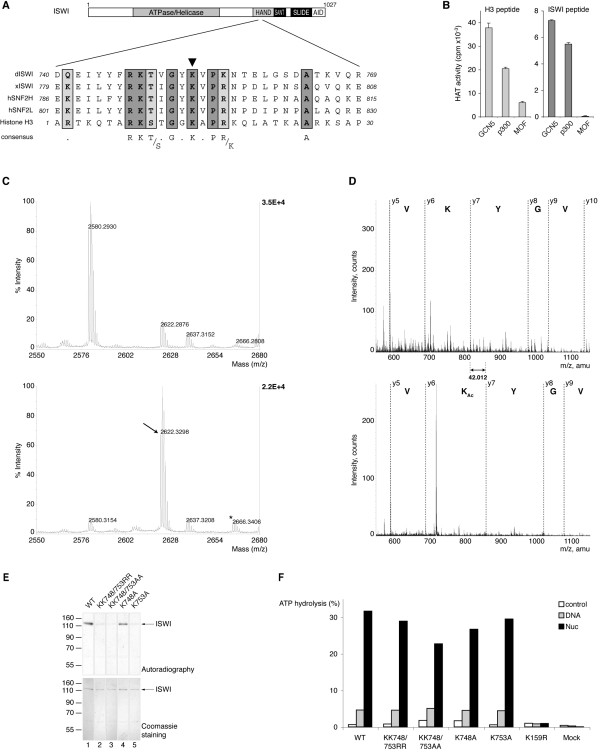
**GCN5 acetylates ISWI at K753**. (A) Alignment of *Drosophila *ISWI sequences spanning aa 740–769 with the corresponding residues from *Xenopus *ISWI (xISWI), human hSNF2H, hSNF2L and with the 30 N-terminal amino acids of histone H3. A consensus of highly conserved residues is given. The arrowhead points to K753 indISWI. (B) Peptides corresponding to the histone H3 N-terminal tail (aa 1–19, left panel) and to aa 740–759 of ISWI (right panel) were acetylated *in vitro *by GCN5, p300 or MOF as in Figure 2. Peptide acetylation was determined from incorporation of [^3^H]-acetate and by subtracting the values for autoacetylation of enzymes (from reactions lacking substrate) and the values for non-specific adsorption of label to the peptides (from reactions lacking enzyme) from the experimental values. (C) MALDI analysis of the *in vitro *acetylated ISWI peptide (aa 740–759). The acetylation reaction was stopped by addition of 10% acetic acid, and the reaction products were analyzed by MALDI-TOF. The upper spectrum represents the unmodified peptide (M-H^+ ^= 2580.29 amu) from a control reaction lacking acetyl-CoA. In the lower spectrum the arrow points to a peak representing the product of the acetylation reaction (M-H^+ ^= 2622.32 amu). A Deltamass (Dm) of 42.01 amu indicates mono-acetylation. A second peak of very low intensity (asterisk) suggests minor di-acetylation (M-H^+ ^= 2664.33). (D) Nanospray sequencing of ISWI peptide (aa 743–759) after *in vitro *acetylation and removal of the C-terminal cysteine by V8 protease. Upper spectrum: sequencing of the unmodified peptide with a (M-3H^+^) value of 702.67 confirms the sequence IYYFRKTVGYKVPKNTE with unmodified K748, K753 and K756. The spectrum is enlarged over the region _750_VGYKV_754 _in order to observe the y7 ion (M-H^+ ^= 815.46) corresponding to the unacetylated K753. Lower spectrum: sequencing of the peptide with a (M-3H^+^) value of 716.73 reveals the sequence IYYFRKTVGYK_Ac_VPKNTE, with only K753 being acetylated, (mass of the y7 ion M-H^+ ^= 857.47, which differs in mass from the corresponding unmodified y7 ion in the upper spectrum by 42.012 amu). Detailed analysis of K748 and K756 revealed no acetylation at these sites (data notshown). (E) Recombinant ISWI and derivatives bearing the indicated point mutations were acetylated by GCN5 and acetylated proteins were visualized by autoradiography (upper panel) and Coomassie staining (lower panel). (F) Recombinant wildtype (WT) and mutant ISWI proteins were incubated with [g-^32^P]-ATP in the absence (control), or presence of 110 ng of free or nucleosomal DNA (Nuc) as indicated. ATP hydrolysis was detected by thin-layer chromatography and quantified by PhosphoImager analysis. ATPase activity is indicated as percentage of ATP hydrolysed.

We next wished to identify the site(s) at which ISWI was acetylated by GCN5 *in vitro*. The ATPase domain of ISWI is located in the central part of the protein (Figure 3A). While more N-terminal sequences of the protein have no discernable features, the structure and function of the C-terminal third are better known. A region defined by HAND, SANT and SLIDE domains harbours a prominent nucleosome recognition module [15]. At the very C-terminus an ACF1-interaction determinant (AID) has been described [16]. A panel of different ISWI fragments fused to hexahistidine- (His_6_-) or GST-tags (Figure 3) were expressed in *E. coli *and used as substrates for GCN5-dependent acetylation. Acetylation of the first 697 N-terminal amino acids (aa) of ISWI containing the ATPase domain and sequences up to the HAND domain was barely detectable (Figure 3B, lane 1). By contrast, the remaining C-terminal part (aa 691–1027) was strongly labelled (Figure 3B, lane 2). Trimming this fragment from the C-terminus to aa 962 did not affect its acetylation, however, deleting amino acids between 691 and 796 abolished the acetylation (Figure 3B, lanes 4–7). We concluded that a prominent acceptor site for acetylation must reside within the HAND fold [15]. This region of ISWI contains 10 lysines that could potentially be acetylated. Upon closer inspection we found the region between aa 747 and 756 to be remarkably similar to the N-terminus of histone H3 (Figure 4A). In this alignment ISWI residues 748 and 753 (K748 and K753, respectively) correspond to lysines 9 and 14 of H3, which are frequently acetylated by GCN5. These ISWI residues and their sequence context are conserved in *Xenopus laevis *(xISWI) and the human ISWI isoforms hSNF2H and hSNF2L (Figure 4A). We therefore focused our attention on this part of ISWI.

A peptide corresponding to aa 1–19 of histone H3 is acetylated rather well by GCN5 and p300 in a standard HAT reaction, but less by MOF (Figure 4B, left panel). By direct comparison, a peptide spanning the corresponding 19 aa of ISWI (Figure 4A) is well acetylated by GCN5, but not at all by MOF (Figure 4B, right panel). Maldi-TOF mass spectrometry and nano-electrospray sequencing documented that the peptide was mono-acetylated (Figure 4C) exclusively at K753 (Figure 4D). In order to test for the contribution of K753 to ISWI acetylation in the context of the entire protein we expressed recombinant ISWI derivatives that had either K748 or K753 or both lysines replaced by alanines (A) or arginines (R). Equivalent protein amounts (Figure 4E, lower panel) were used as substrate for GCN5-dependent acetylation reactions. The ISWI derivative bearing the K748A mutation was acetylated as the wildtype protein, whereas replacement of lysine 753 by alanine (K753A) led to a significantly reduced acetylation (Figure 4E, compare lanes 4, 5). Collectively the data identify K753 of ISWI, which appears related by sequence context to H3K14, as the major target of acetylation by GCN5 *in vitro*.

We were not able to evaluate the effect of acetylation on ISWI ATPase activity due to the small fraction of ISWI modified in these *in vitro *reactions. However, replacement of either lysine 753 or lysine 748 (which corresponds to lysine 9 in H3) by arginine or alanine did not affect the ATPase activity in response to DNA or nucleosomes (Figure 4F).

### ISWI is acetylated at K753 by GCN5 in vivo

**Figure 5:**
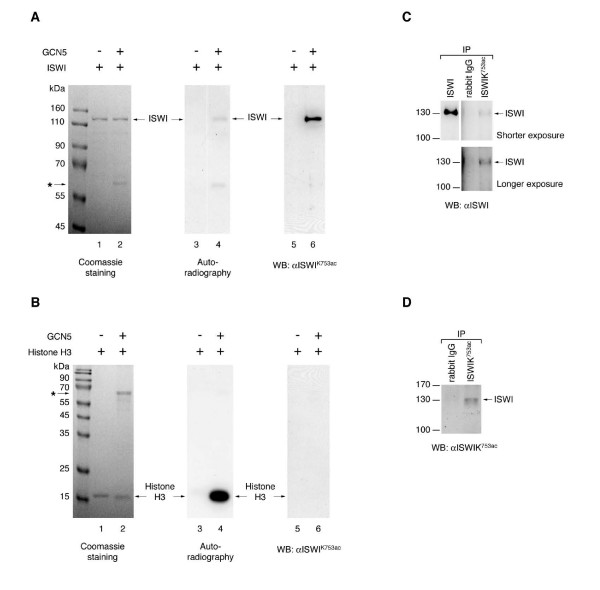
**Characterisation of an antibody specific forISWI^K753ac^**. (A) Recombinant ISWI was acetylated by GCN5 in the presence of radiolabeled acetyl-CoA. The protein mixture was separated by SDS-PAGE and visualized by Coomassie staining (left panel), by autoradiography (centre panel) and by Western blotting using an antibody raised against an ISWI peptide bearing acetylated K753 (αISWI^K753ac^). The asterisk points to the position of GCN5 in the gel (lane 2), which autoacetylates itself (lane 4). (B) The antibody does not cross-react with acetylated H3. Recombinant histone H3 was acetylated with GCN5 in presence of radiolabeled acetyl-CoA and analysed as in (A). Western blotting on such a substrate using αISWI^K753ac ^did not reveal any cross-reactivity. (C) ISWI was immunoprecipitated from embryonic nuclear extract using antibodies against ISWI or ISWI^K753ac ^and detected by Western blotting. (D) αISWI^K753ac ^immunoprecipitates were analysed by Western blotting for the presence of acetylated ISWI.

In order to monitor the distribution of ISWI^K753ac ^*in vivo *we raised an antibody against an ISWI peptide acetylated at K753. The antibody recognised ISWI only after acetylation by GCN5 (Figure 5A, lanes 5, 6) and did not cross-react with H3 even when the histone was acetylated to much higher levels (Figure 5A, B; compare lanes 4 and 6). The antibody precipitated ISWI from *Drosophila *embryo extracts (Figure 5C) as well as a protein of the size of ISWI that was stainable with the ISWI^K753ac ^antibody (Figure 5D).

**Figure 6:**
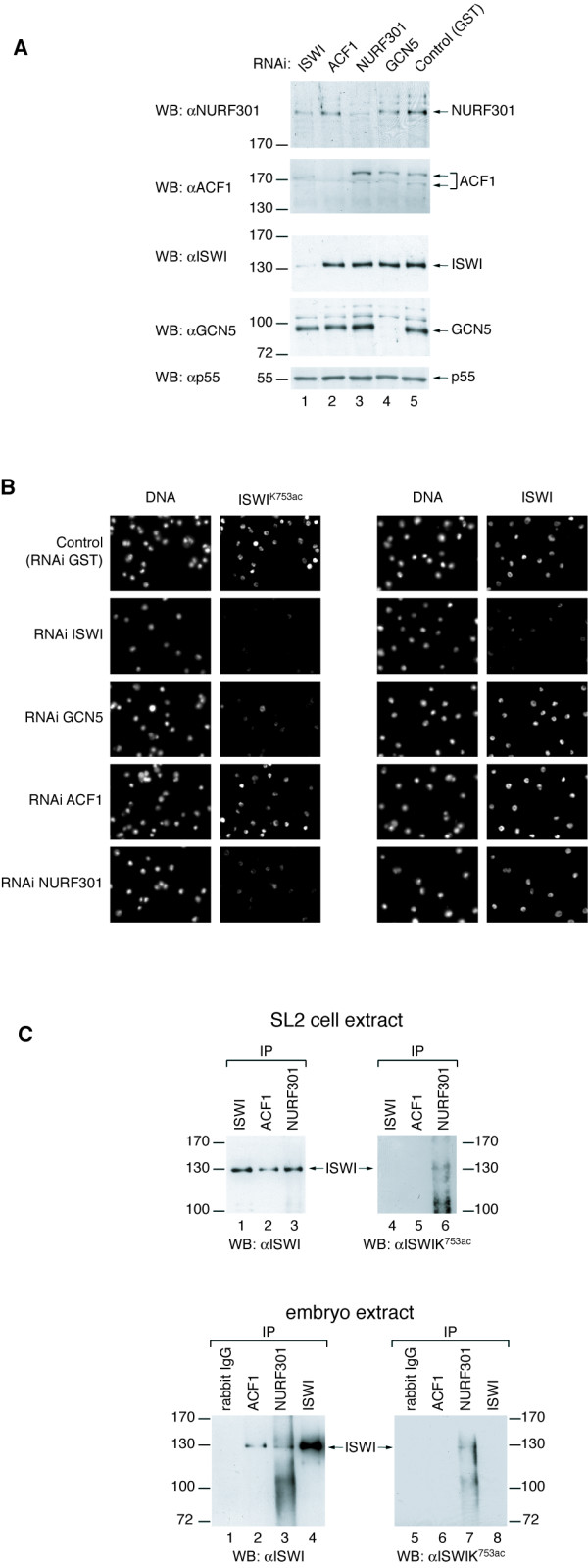
**Acetylated ISWI may reside in the NURF complex, but not in CHRAC/ACF in SL2 cells**. (A) Ablation of proteins by RNA interference. *Drosophila *SL2 cells were treated with double-stranded RNA (RNAi) to interfere with the expression of ISWI, ACF1, NURF301, GCN5 or GST (to control for non-specific effects of dsRNA treatment). After 10 days of incubation whole cell lysates were prepared and probed by Western blotting (WB) for the presence of NURF301, ACF1, ISWI, GCN5 and p55 (a subunit of NURF that serves as a loading control). (B) SL2 cells were treated for 12 days with dsRNAs as indicated. Localization of ISWI^K753ac ^or bulk ISWI was monitored by immunofluorescence staining using the indicated antibodies. DNA was counterstained with Hoechst 33258. (C) SL2 whole cell extract (upper panel) or nuclear extract from 8–12 hr embryos (lower panel) were subjected to immunoprecipitation with antibodies against ISWI, ACF1 or NURF301. The presence of bulk ISWI (left panel) or ISWI^K753ac ^(right panel) in the immunoprecipitates was analysed by Western blotting.

The antibody also recognised an epitope in nuclei of *Drosophila *SL2 cells (Figure 6B, upper left panel). To confirm that the antibody reactivity was specific *in vivo *we performed RNA interference on SL2 cells with dsRNA corresponding to ISWI or GCN5 sequences and monitored the success of the knockdown by Western blotting of whole cells extracts (Figure 6A) and immunofluorescence (Figure 6B). Knocking down either ISWI or GCN5 slowed cell proliferation effectively increasing the doubling time two fold. Ablation of ISWI removed not only the signal obtained with the ISWI antibody, but also the ISWI^K753ac^-specific signal (Figure 6B). Remarkably, knockdown of GCN5 also greatly reduced the signal obtained with the ISWI^K753ac ^antibody, but did not affect ISWI in general (Figure 6B). Taken together, these data document the specificity of the antibody for ISWI^K753ac ^in immunofluorescence applications. They also show that ISWI is acetylated at K753 by GCN5 in SL2 cells.

### Acetylated ISWI may reside in the NURF complex, but not in CHRAC/ACF

So far ISWI is known to reside in two kinds of nucleosome remodelling complexes in *Drosophila*: CHRAC/ACF, which are characterised by the ACF1 subunit, and NURF, which contains NURF301 as a defining feature [17]. In order to explore whether ISWI^K753ac ^was preferentially associated with one of these complexes we ablated the signature subunits ACF1 and NURF301 by RNA interference in SL2 cells (Figure 6A) and found that the ISWI^K753ac ^epitope was significantly reduced in the absence of NURF301, but remained unperturbed if ACF1 was knocked down (Figure 6B). In agreement with the notion that ISWI^K753ac ^is not a subunit of CHRAC/ACF we found that the expression pattern of the acetylated ISWI form was unperturbed in *acf1 *null flies (data not shown). ISWI^K753ac ^staining was unperturbed in flies heterozygous for the NURF301 allele [E(bx)^ry122^] [18]. Homozygous mutants are lethal, but survive until the end of the larval stage due to maternal contribution of NURF301 and were not analysed. Immunoprecipitation of NURF301 from extracts of SL2 cells and 8–12 hr embryos co-precipitated acetylated ISWI, which was not the case if ACF1 was precipitated (Figure 6C, D). We conclude that in SL2 cells ISWI may be acetylated in the context of NURF but is not acetylated in association with ACF1. The data do not exclude the existence of additional complexes harbouring the modification.

## Discussion

Many acetyltransferases were first identified following their histone acetylation function and hence named histone acetyltransferases (HATs). However, most of them have now been shown to acetylate non-histone proteins as well, and these modifications can have profound effects on the structure and function [19]. For example, the effects of acetylation on the tumour suppressor protein p53 have been heavily studied [20]. The current work, together with the earlier observation that the effect of the BRM ATPase on cell proliferation can be regulated by acetylation [10] highlight the potential for direct regulatory cross-talk between different chromatin modifying principles [21].

The fact that ISWI can be acetylated *in vitro *and in tissue culture cells by GCN5, which is also known to target the H3 N-terminus, is explained by the sequence similarity between the histone and ISWI around the K753 acetylation site. The common motif RK^T^/_S_xGx(K^ac^)xP^R^/_K _differs from the previously suggested GCN5/PCAF recognition motif GKxxP [19]. This raises the possibility of specific co-regulation of a nucleosome remodelling factor and its nucleosome substrate through acetylation of related epitopes. Strikingly, the related motif DKGKGKKRP also occurs in the C-terminus of hBRM, overlapping with potential acetylation sites [10]. Acetylation at one or more C-terminal lysines of BRM correlates with reduced interference with cell cycle progression in mammalian cells and mutation of several of these lysines into arginines impairs BRM function. The data suggest that site-specific acetylation of the ATPase by, for example P/CAF, may adversely affect its function, possibly by interfering with targeting interactions [10].

The fact that the acetylated ISWI epitope is conserved during evolution suggests functional significance. However, so far we were unable to document an effect of acetylation on the catalytic properties or protein interactions of ISWI, due to the small proportion of ISWI either acetylated *in vitro *or naturally present *in vivo*. Lysine 753 of ISWI resides in the HAND domain, a fold of unknown function situated between the ATPase domain and the C-terminal SANT/SLIDE domains that mediate nucleosome interactions [15]. Exploring the role of the HAND domain in ISWI may lead to novel approaches towards evaluating the impact of K753 acetylation. Applying the K753ac-specific antibody to developmental stages of *Drosophila *revealed that the acetylation of ISWI is a developmentally regulated process, which occurs during oogenesis and early embryogenesis, but is absent from later developmental stages (CC and PBB, unpublished observations). We also obtained preliminary support for the idea that the acetylation of ISWI marks a functionally distinct subpopulation: the acetylated epitope was dramatically enriched on the condensed metaphase chromosomes in early embryos (CC and PBB, unpublished observations). A causal role between the acetylation and the unusual ability of the ATPase to interact with compact chromatin remains to be explored.

The ATPase ISWI is the catalytic moiety of several nucleosome remodelling complexes. Better known and characterized are the factors CHRAC, ACF and NURF (for review, see [22]. The available data suggest that the acetylated form of ISWI may mark a subpopulation of the NURF complex. NURF is involved in the transcription of selected sets of *Drosophila *genes [18,23] but flies mutant for the large, defining NURF301 subunit also show defects of chromosome structure [23]. Assessment of which of these functions may be modulated by ISWI acetylation requires a genetic approach.

The acetylation of the amino terminus of histone H3 is an important determinant of active chromatin, but chromatin structure can also be modulated by other modifications of the histone 'tail', notable its methylation or phosphorylation. Whether the analogy between the ISWI and the H3 N-terminus can also be extended to include phosphorylation and methylation remains an interesting aspect for future research.

## Conclusion

We identified and characterised a site-specific acetylation of the remodelling ATPase ISWI by GCN5. The modification occurs predominantly in the context of the remodelling factor NURF, however, due to the fact that the acetylation marks a minor subpopulation of the remodeller a functional evaluation of the modification has not yet been achieved.

## Methods

### Recombinant DNA technology

pGEX-4T-3 and pProEx-Htb vectors for expression of GST-ISWI and (His6)-ISWI deletion mutants, respectively have been described earlier [15,16]. pFastBacHTa-FLAG-ISWI WT was generated by inserting the NcoI-KpnI fragment from pMYB4-FLAG-ISWI WT [24] into pFastBacHTa vector ("Bac-to-Bac" expression system, Invitrogen). pMYB4-FLAG-ISWIK748A, -ISWIK753A, -ISWIKK748/753AA and -ISWIKK748/753RR were generated using the QuickChange site-directed mutagenesis kit (Stratagene) with primers listed in the supplemental materials and moved as NcoI-KpnI fragments into the pFastBacHTa vector.

### Primers used for site-directed mutagenesis

Generation of ISWI^K748A^:

CCAGGAAATCTACTATTTCCGGGCCACTGTTGGTTAC and

GTAACCAACAGTGGCCCGGAAATAGTAGATTTCCTGG.

Generation of ISWI^K753A^:

GTTGGTTACGCGGTACCCAAGAACACGGAACTAGG and

CCTAGTTCCGTGTTCTTGGGTACCGCGTAACCAAC.

Generation of ISWI^K748R^:

CCAGGAAATCTACTATTTCCGGAGGACCGTTGGTTAC and

GTAACCAACGGTCCTCCGGAAATAGTAGATTTCCTGG.

Generation of ISWI^K753R^

GTTGGTTACCGAGTGCCCAAGAACACGGAACTAGG and

CCTAGTTCCGTGTTCTTGGGCACTCGGTAACCAAC

### Primers used for PCR to generate dsRNA fragments for RNA interference

ISWI sequences 1–499:

TTAATACGACTCACTATAGGGAGAATGTCCAAAACAGATACAGCTG and

TTAATACGACTCACTATAGGGAGAGCAGAGATATGGTCTGCAGG;

GCN5 sequences 1–525:

TTAATACGACTCACTATAGGGAGAATGTCTGGTGGTCCATCC and

TTAATACGACTCACTATAGGGAGAGCGCTGCATGGACATGAA;

ACF1 sequences 1–560

TTAATACGACTCACTATAGGGAGAATGCCCATTTGCAAGCGG and

TTAATACGACTCACTATAGGGAGAATGCGCGAGCGACGTAAT;

NURF301 sequences 1–521

TTAATACGACTCACTATAGGGAGAATGAGCGGTCGCGGCAG and

TTAATACGACTCACTATAGGGAGAGCTGCATACTGACGACCT;

GST sequences 1–527;

TTAATACGACTCACTATAGGGAGAATGTCCCCTATACTAGGTTA and

TTAATACGACTCACTATAGGGAGAACGCATCCAGGCACATTG;

### Antibodies

The following antibodies were kind gifts: αISWI (J. Tamkun, UC St. Cruz), αp55 (C. Wu, NIH, Bethesda), αGCN5 (Jerry Workman, Stowers Institute for Medical Research) αNURF301 (Andreas Hochheimer). The monoclonal αACF1 antibody was raised in rats. Its specificity was confirmed using the *acf1 *null fly strain [25] (C. Chioda et al, manuscript in preparation). We employed two different pan-acetyl-lysine antibodies for immunoprecipitation [26] and Western blotting (Cell Signalling Technology # 9441). The αISWI^K753ac ^was raised against ISWI peptide TVGYKacVPKNT (aa 749–758) where K753 was acetylated. The antibody is available from Abcam (ab10748). As control antibody we used rabbit IgG (sc-2027, Santa Cruz Biotechnology). The αH3^S10p ^antibody was from Upstate (RR002, catalog # 05–598).

### Cell culture, RNA interference and immunostaining

*Drosophila *SF4 cells (an SL2 clone sorted for diploidy and adherence) were provided by D. Arndt-Jovin (Göttingen). SF4, Kc and SL2 cells were maintained at 26°C in Schneider's Drosophila medium (Invitrogen) supplemented with 10% fetal calf serum (FCS), antibiotics and glutamine. When needed, trichostatine A (TSA) was added at a final concentration of 200 ng/ml to the medium for 17 hrs before harvesting the cells. RNAi experiments were performed as described [27]. The primers used to generate the double-stranded RNA are listed in the supplemental materials. Briefly, 1–2 × 106 SL2 cells were seeded into 6-wells plates in 1 ml of medium without FCS just before adding 10 μg of dsRNA. Plates were then placed onto a shaking platform for 10 min and then for 50 min at 26°C. 2 ml of medium complemented with FCS were then added to the cells and incubated at 26°C. Cells were collected 10 or 12 days after dsRNA treatment. Protein extractions for Western blot analysis after RNAi, 1–2 × 106 cells were pelleted and lysed in 50 μl of urea buffer (8 M urea, 5% SDS, 200 mM Tris-Cl pH 6.8, 0.1 mM EDTA, 100 mM DTT) and incubated at 65°C for 15 min. Immunostainings were performed as described [28]. For immunostaining following RNAi, cells were collected 12 days after dsRNA treatment. After fixation, permeabilisation and blocking, cells were incubated for 1 hr with antibody against ISWI, diluted 1:600 in blocking solution (2% BSA and 5% goat serum in PBS), or aISWIK753ac, diluted 1:300. After washes, cells were incubated for 1 hr with Cy3-conjugated secondary antibody (Jackson Immunoresearch Laboratories) diluted in blocking buffer. Cells were washed four times in PBS. DNA was counterstained with 1 μg/ml bisbenzimide (Hoechst 33258). Slides were mounted using 1.5% n-propyl gallate, 50% glycerol in PBS. Images were acquired using a Zeiss Axiophot microscope coupled to a Retiga Exi CCD Camera (Qimaging, Burnaby, Canada). Images were cropped and levels adjusted in Photoshop.

### Immunoprecipitation

Total cell extracts were prepared as described [29] from 4 × 10^7 ^Drosophila cells. All steps were performed at 4°C. Cells were washed with PBS, resuspended in 1 volume of lysis buffer (50 mM Tris-Cl pH 8.0, 300 mM NaCl, 10 mM MgCl2, 0.4% NP40 and proteases inhibitors) and incubated for 15 min. The supernatant was cleared by centrifugation and mixed with 1 volume of dilution buffer (50 mM Tris-Cl pH 8.0, 0.4% NP40). Diluted extracts were pre-cleared with protein A/protein G Sepharose beads (Amersham Biosciences) and incubated with αISWI, αAcLysine or irrelevant antibodies, and immunoprecipitated with protein A/protein G Sepharose beads. Following extensive washing with a 1:1 mix of lysis and dilution buffer, beads were resuspended in Laemmli buffer and proteins were analyzed by Western blot analysis.

### Western Blot Analysis

Immunoprecipitated proteins were separated by SDS-PAGE, electro-transferred onto PVDF or nitrocellulose membranes and detected using an ECL kit (Amersham Biosciences) according to the manufacturer's instructions.

### In vivo labeling

*Drosophila *cells were treated with 0.5 mCi/ml [^3^H]-acetic acid (TRK12, Amersham Biosciences) and 10 mM sodium butyrate (NaB) for 3 hrs at 26°C. Whole cell extracts and immunoprecipitations were performed using standard procedures, except that all buffers were complemented with 5 mM sodium butyrate. Immunoprecipitated proteins were analyzed by Western blot or by autoradiography.

### Recombinant proteins

FLAG-ISWI mutants were expressed and purified from baculovirus vectors in Sf9 cells as described [30]. Expression of proteins in *E. coli *and purification was according to the following published procedures: FLAG-ISWI and HIS_6_-ISWI deletion mutants [15]; GST-ISWI deletion mutants [16]; GST-hGCN5 and FLAG-p300 [31]; MOF [13].

### In vitro acetylation assays

200 ng of FLAG-ISWI, 200 ng of His- or GST-ISWI deletion mutants, 2 μg of histone octamers or 200 ng of bacterially expressed histone H3 were incubated for 30 min at 26°C, in 20 μl final volume with 0.25 mCi of [^3^H]-acetyl-CoA (4.1 Ci/mmol, TRK688, Amersham Biosciences), 50 to 100 ng of GST-hGCN5, HA-MOF or FLAG-p300 in HAT buffer (10 mM Tris-Cl pH 7.8, 0.1 mM EDTA, complemented with 1 mM PMSF, 1 mM DTT and 10 mM NaB). Reaction mixtures were analyzed by SDS-PAGE. The gel was Coomassie stained, destained, treated with Amplify (Amersham Biosciences) for 30 min, dried and autoradiographed. 1 μg each of an H3 peptide (aa 1–19) or an ISWI peptide (aa 740–759) were acetylated under the same conditions. A 10 μl aliquot of each reaction was spotted onto p81 filters (Whatman), which were washed three times with 50 mM NaCarbonate pH 9.2 and counted in a scintillation counter.

### Sequence alignment

Sequence alignment was performed using ClustalW software, using default parameters.

### Mass spectrometry

*MALDI-TOF analysis of acetylated ISWI peptide*. The *in vitro *acetylated peptides were purified on C18 reversed phase ZipTip mini-columns (Millipore) according to the manufacturer's protocol. In short the peptide was washed 3 times with 0.1% TFA, eluted with 1 μl of matrix solution [saturated a-cyanohydroxy-cinammic acid (Sigma) dissolved in 50% ACN (v/v)/0.3% TFA (v/v)] directly onto the target plate. The peptide-matrix co-crystal was analyzed in a Voyager DE STR spectrometer according to the manufacturer's instructions. Peptide mass fingerprints covered the mass range between 700–3500 amu, with the low mass gate set at 500 amu. The accelerating voltage was set to 20 kV, the grid to 66% and the delay time to 100 nsec. *ESI-analysis*. The ISWI peptide DQEIYYFRKTVGYKVPKNTEC plus a C-terminal cysteine (aa 740–759, M-H^+ ^= 2580,27 amu) was digested with V8 protease. The resulting peptide IYYFRKTVGYKVPKNTE (M-H^+ ^= 2106.14 amu) was de-salted, concentrated using a C18 reversed phase minicolumn (Eppendorf) and eluted in 0.5 μl 50% Methanol, 0.1% FA into medium size nano-spray needles (Protana, Odense, Denmark). ESI mass spectra were recorded on an Applied Biosystems QStar XL hybrid quadrupole time of flight mass spectrometer, equipped with a Protana nano-spray ion source in the static nano-spray mode according to the manufacturer's instructions. The needles were adjusted in front of the orifice and the spray voltage was set between 950 and 1100 V. Product ion scans were acquired for 4–5 min containing approximately 200–300 scans.

### ATPase assays

The ATPase assays were performed as described previously [30].

## Authors' contributions

RF discovered ISWI acetylation and did most of the biochemical analysis, prepared the figures and contributed to preparing the manuscript. AE expressed recombinant ACF in vitro, contributed the ATPase assay and commented on the manuscript. CC performed experiments not shown aimed at revealing a function of ISWI acetylation in tissue culture cells and helped prepare the manuscript. TB performed the mass spectrometrical analysis. AI contributed to interpretation of the mass spectometrical analysis. PB coordinated the project, provided funds and wrote the manuscript.
